# The direct and mediating effect of social support on health-related quality of life during pregnancy among Australian women

**DOI:** 10.1186/s12884-023-05708-0

**Published:** 2023-05-22

**Authors:** Asres Bedaso, Jon Adams, Wenbo Peng, David Sibbritt

**Affiliations:** 1 College of Medicine and Health Sciences, School of Nursing, Hawassa University, Hawassa, Ethiopia; 2 School of Public Health, Faculty of Health, University of Technology Sydney, Ultimo, NSW Australia

**Keywords:** Health-related quality of life, Social support, Stress, Pregnancy, Mediation

## Abstract

**Background:**

Prenatal stress can have a negative effect on the quality of life (QoL) of pregnant women. Social support plays a vital role in improving the psychological well-being of pregnant women by enhancing their stress-coping ability. The current study assessed the association between social support and health-related quality of life (HRQoL) as well as the mediating role of social support in the linkage between perceived stress and HRQoL among pregnant Australian women.

**Methods:**

Secondary data was obtained from 493 women who reported being pregnant in survey six of the 1973–78 cohort of the Australian Longitudinal Study on Women’s Health (ALSWH). Social support and perceived stress were assessed using the Medical Outcomes Study Social Support Index (MOS-SSS-19) and the Perceived Stress Scale, respectively. The Mental Component Scale (MCS) and Physical Component Scale (PCS) of the SF-36 were used to examine the mental and physical HRQoL. A mediation model was used to examine the mediating effect of social support in the relationship between perceived stress and HRQoL. A multivariate quantile regression (QR) model was used to assess the association between social support and HRQoL after adjusting for potential confounders.

**Result:**

The mean age of the pregnant women was 35.8 years. The mediational analysis revealed that emotional/informational support (β= **-**1.53; 95% CI: -2.36, -0.78), tangible support (β= -0.64; 95% CI: -1.29, -0.09), and affectionate support/positive social interaction (β= **-**1.33; 95% CI: -2.25, -0.48), played a significant mediating role in the relationship between perceived stress and mental health-related QoL. In addition, perceived stress had a significant indirect effect on mental health-related QoL through overall social support (β = -1.38; 95% CI: -2.28, -0.56), and the mediator accounted for approximately 14.3% of the total effect. The multivariate QR analysis indicated that all the domains of social support and overall social support scores were positively associated with higher MCS scores (p < 0.05). However, social support was found to have no significant association with PCS (p > 0.05).

**Conclusion:**

Social support plays a direct and mediating role in improving the HRQoL of pregnant Australian women. Maternal health professionals need to consider social support as an essential tool to improve the HRQoL of pregnant women. Further, as part of routine antenatal care activity, assessing pregnant women’s level of social support is beneficial.

**Supplementary Information:**

The online version contains supplementary material available at 10.1186/s12884-023-05708-0.

## Background

Pregnancy can be a stressful time for most women [1, 2]. The occurrence of hormonal and physiological changes [3] and stressful events during pregnancy could negatively affect the physical and mental health of pregnant women, and as a consequence, their quality of life (QoL) will worsen [4]. QoL is defined as an “individual’s perception of their physical and mental health, level of independence, social relationships, personal beliefs, as well as their relationships to their environment” [5].

HRQoL is a sub-component of QoL and is defined as a value given to the duration of life as altered by one’s functioning ability and disability, perceptions, and social opportunities as a result of body changes, illness, injury, or treatment [6]. Studies have shown that poor HRQoL during pregnancy resulted in low birth-weight infants [7, 8], higher gestational weight gain [9], experiencing symptoms such as fatigue, back and pelvic pain [10] and low HRQoL in the postnatal period [11]. Stress is commonly experienced by pregnant women [12], which can have an adverse impact on HRQoL [13, 14].

Epidemiological data indicated that the prevalence of stress during pregnancy is 12-36.1% in Canada [15, 16], 78% in the US [12] and 95% in China [17]. A prospective study examining the trend of prenatal stress among Australian women reported the highest prevalence of stress in the early and late stages of pregnancy [18]. It has been suggested that stress could exacerbate gestational diabetes mellitus and preeclampsia, which adversely affect the HRQoL of pregnant women [19, 20]. In addition, a pregnant woman’s concerns about her body image and increased weight gain could also contribute to the risk of developing mental health problems such as antenatal anxiety and depression [21, 22], which could lead to poor HRQoL [23, 24].

Factors such as being pregnant at a young age [25], low socioeconomic status [26], unplanned pregnancy, poor self-care, no antenatal care [27], and third trimester of pregnancy [28] have all been associated with poor HRQoL during pregnancy. However, women undertaking the recommended level of physical activity during pregnancy [29], low-parity pregnant women [26, 30, 31] and those with first and second trimesters of pregnancy [8, 32] were strongly related to a better HRQoL. Further, a systematic review has revealed that high social support and less perceived stress are associated with improved HRQoL during pregnancy [33].

Social support refers to the provision of emotional, informational, affectionate, and tangible support for somebody through the available social network [34]. Followup studies conducted on perinatal women have reported that providing social support can decrease stress and increase the likelihood of recovery, thereby improving the HRQoL [35, 36]. It has been suggested that social support interventions and social participation are effective in preventing prenatal and neonatal adverse birth outcomes by minimising the impact of stress on the mental and physical well-being of pregnant women [37, 38]. Social support is also proven highly effective for acute care during life crises [39] and subjective assessment of health states [40].

Different models have suggested social support as an area for intervention to improve the HRQoL of pregnant women. The stress-buffering hypothesis suggests that social support could mediate the relationship between perceived stress and HRQoL during pregnancy [41]. Mainly, social support improves the HRQoL of individuals by enhancing their positive affect and stress coping ability [42]. Studies have also demonstrated that social support directly affects individuals’ HRQoL (i.e., those with less social support have a lower HRQoL than those with higher social support), irrespective of their stress level [43, 44].

However, there is limited evidence reporting the effect of social support on HRQoL among pregnant women. In response to this research gap, our study aimed to examine the direct effect of social support on HRQoL and its mediating role in the linkage between perceived stress and HRQoL.

## Method

### Data source

This study analysed data from the 1973–78 cohort of the Australian Longitudinal Study on Women’s Health (ALSWH) [45, 46] and reported per the STROBE guideline (Supplementary file 1). The ALSWH is an ongoing community-based longitudinal study focusing on the health and well-being of Australian women. Over 40,000 women were recruited to participate in 1996 (baseline survey) in three age cohorts (birth year: 1973–78, 1946–51 and 1921–26). Participants were selected randomly via the national health insurance database. Of the 8,010 women who completed Survey 6 of the 1973–78 cohort in 2012 (age between 34 and 39 years), those who reported being pregnant (n = 493) were included in the current analyses [47].

### Measurement

The Medical Outcomes Study Social Support index (MOS-SSS-19) was used to examine social support given to pregnant women. The MOS-SSS-19 has an overall index of 19 items (Cronbach’s alpha 0.81), with higher scores indicating greater social support. The MOS-SSS-19 has three functional support subscales: emotional/informational support, tangible support, and affectionate support/positive social interaction [48].

The level of stress in the past 12 months was assessed using the Perceived Stress Questionnaire [49]. The tool measures the level of perceived stress in specific areas, such as relationships and own health. An overall mean stress score ranges from 0 (no stress) to 4 (extreme stress). The tool has good internal reliability (α = 0.75) [50, 51].

The Mental Component Scale (MCS) and Physical Component Scale (PCS) of the SF-36 [52] were used to examine the mental and physical HRQoL of pregnant women, with higher scores indicating a better QoL. Scores were standardised using Australian norms to get a mean of 50 and a standard deviation of 10 [53].

### Statistical analysis

The statistical software package SPSS Statistics 26.0 was used for all analyses. The comparison of the PCS and MCS scores between different participant groups was conducted using a t-test and one-way ANOVA. Pearson correlation coefficient was determined to test the relationships between perceived stress, social support, and components of HRQoL (MCS and PCS).

**Fig. 1:**
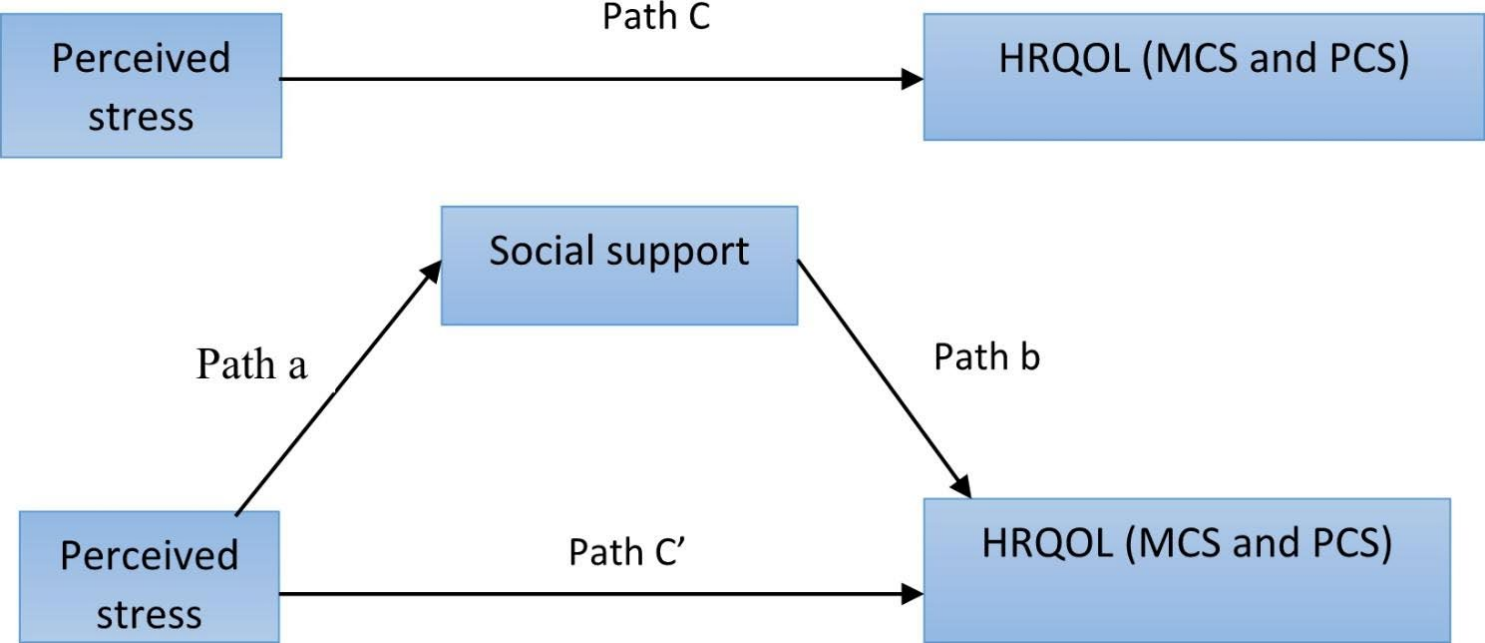
The proposed mediation model to examine the mediating role of social support

The mediating effect of social support in the linkage between stress and components of HRQoL was examined using the PROCESS macro (version 3.0) for SPSS [54]. A 3-step regression-based analysis was performed to test the mediational role of social support in the relationship between stress and HRQOL (i.e. MCS and PCS). Coefficients for each path (a, b, c, and c’) in the mediation model were displayed in Fig. 1. In the first step, the overall social support score and each domain of social support were regressed on perceived stress (path a). In the second step, HRQoL (i.e. MCS and PCS) was regressed on domains of social support and overall social support (path b). In the third step, the outcome variables (i.e. MCS and PCS) regressed on stress (path c’). Path c’, displays the linkage between the independent variable (perceived stress) and the outcome variable (MCS and PCS) by excluding the mediator (social support), whereas path c (total effect) with the inclusion of the mediator. Separate mediation analyses were conducted by considering each domain of social support and overall MOSS-SSS score as a mediator to examine whether there is variation in the mediating effects of each domain of social support.

The mediation effect (indirect effect) (c-c’=a*b) of social support is declared when there is a statistically significant difference between path c and path c’ [55]. The total effect (path c), indirect effects (path a*b) and direct effects (path c’) were reported in the form of unstandardised beta coefficients (${\beta }$). The bootstrapping procedures in the SPSS PROCESS macro from the mediation model 4 were used to test the significance of the indirect effects of perceived stress on HRQoL (i.e. MCS and PCS) through the mediation of social support [56]. During our mediational analysis, heteroscedasticity consistent standard error and adjusted covariance matrix estimator (HC2) were considered to adjust for the abnormal error distribution in the outcome variable [57].

The percent mediation (P_M_ = a*b/c) and R-squared mediation (R^2^ med) were also determined. P_M_ is the ratio of the indirect effect to the total effect and can be interpreted as the percent of the total effect accounted for by the indirect effect [58, 59]. R^2^ med is the variance of the outcome variable (i.e. MCS and PCS) and can only be explained by both the independent variable (perceived stress) and mediator (social support) [60].

The direct effects model states that social support is directly related to its outcome without involving any intermediate variable [61]. For example, evidence has shown that social support has a direct effect on the quality of life of individuals, irrespective of their level of stress [43, 44]. Due to the negatively skewed distribution of both the PCS and MCS, we fitted a multivariate quantile regression (QR) model to examine the direct effect of social support on HRQoL (i.e. PCS and MCS) after adjusting for potential confounders [62].

The association between social support and HRQoL was examined at the 25th, 50th, 75th and 90th quantiles. Regression coefficients for each quantile and the corresponding 95% confidence interval (CI) were computed. The adjusted QR controlled for the available potential confounders to examine the association between social support and HRQoL (i.e. MCS and PCS). The association was considered statistically significant at a p-value ≤ 0.05.

## Result

The socio-demographic characteristics of study participants were presented in Table 1. Of the 493 study participants, 468 (95.1%) were partnered, while 319 (65%) attained a university degree. The majority of these women, 330 (67.2%), responded that it is easy to manage on income available. The mean (Standard Deviation) age of the women was 35.8 (1.4) years. Regarding their gestational age, 42% of women were in the last trimester of their pregnancy.

**Table 1:** Socio-demographic characteristics of study participants and result of bivariate analyses (n = 493)

Variables	n (%)	MCS		*P*-value	PCS		*P*-value
		Mean	SD		Mean	SD	
Pregnancy months							
< 3 month	101 (20.5)	49.5	6.2	0.001	52.9	6.8	< 0.001
3–6 month	185 (37.5)	49.3	8.9		48.2	8.9	
> 6 month	207 (42)	52.0	7.9		42.5	9.6	
Highest qualification							
University	319 (65)	50.9	7.6	0.104	46.3	9.6	0.175
Certificate/diploma or trade/apprenticeship	112 (22.8)	50.2	8.4		47.1	10.0	
School only	60 (12.2)	48.5	9.7		48.7	9.7	
Marital status							
Partnered	468 (95.1)	50.7	7.9	0.014	46.6	9.6	0.039
Non-partnered	24 (4.9)	46.5	9.4		50.7	10.7	
Able to manage on income available							
Impossible/Difficult all of the time	43 (8.8)	43.8	10.3	< 0.001	46.4	9.6	0.844
Difficult some of the time	118 (24)	49.8	8.9		46.45	9.6	
Not too bad/It is easy	330 (67.2)	51.6	6.9		46.97	9.7	
Living with one’s own children							
Yes	336 (68.3)	50.2	8.4	0.217	46.45	9.8	0.290
No	156 (31.7)	51.1	7.3		47.45	9.51	

Results from the bivariate analyses revealed that marital status and pregnancy trimester were significantly related to both MCS and PCS (Table 1). Partnered pregnant women reported a higher score of MCS (p = 0.014) and a lower score of PCS (p = 0.039) than non-partnered women. Also, pregnant women who were in the last trimester of their pregnancy reported a higher score of MCS (p = 0.001) and a lower score of PCS (p < 0.001). The ability to manage available income was significantly associated with the MCS, and pregnant women who can easily manage on income available presented a higher MCS score (p < 0.001). Note that the mean and median of domains of HRQoL are shown in Supplementary file 2.

### Correlations between variables

The result of the correlation analysis was presented in Table 2. A significant correlation between the independent, mediating and dependent variables is a prerequisite to conducting a mediational analysis. Perceived stress was negatively associated with emotional/informational support (*r*= -0.39, p < 0.01), affectionate support/positive social interaction (*r*= -0.43, p < 0.01), tangible support (*r*= -0.32, p < 0.01), overall social support (MOS-SSS) (*r*= -0.41, p < 0.01), MCS (r= -0.51, p < 0.01) and PCS (r= -0.14, p < 0.01). MCS has a positive association with emotional/informational support (r = 0.381, p < 0.01), affectionate support/positive social interaction (*r* = 0.35, p < 0.01) and tangible support (*r* = 0.26, p < 0.01). However, the PCS has no significant correlation with any of the domains of social support, as well as the overall social support score, which fails to meet the assumptions for mediational analysis. Therefore, these correlations only support the assessment of the mediating role of social support in the linkage between perceived stress and MCS.

**Table 2:** Correlations among perceived stress, social support, and QoL (MCS and PCS) (n = 493)

	Mean (SD)	Perceived stress	MCS	PCS
MOS-SSS	4.36 (0.71)	-0.411^**^	0.364^**^	0.054
Emotional/Informational support	4.35 (0.78)	-0.398^**^	0.381^**^	0.053
Affectionate support/positive social interaction	4.50 (0.66)	-0.433^**^	0.358^**^	0.069
Tangible support	4.22 (0.82)	-0.321^**^	0.268^**^	0.031

Note: **correlation coefficient is significant at the p < 0.01 level

Abbreviation: MCS: Mental Component score, PCS: Physical Component Score

### The mediating effect of social support

The findings of the mediational analysis are shown in Table 3. Increased overall social support was significantly associated with decreased perceived stress (β = -0.65; 95% CI: -0.83, -0.48) and improved HRQoL (MCS) (β = 2.10; 95% CI: [0.89, 3.32). Perceived stress significantly influenced HRQoL-mental health (MCS) (β = -9.61; 95% CI: -11.25, -7.97), and this relationship was still statistically significant after considering overall social support as a mediator in the model (β = -8.23; 95% CI: -10.05, -6.41).

**Table 3:** Social support mediates the relationship between perceived stress and QoL (MCS) (n = 493)

	*Path a*	*Path b*	*Path c*	*Path c’*	*Indirect effect (β)*	P_M_	R^2^_Med_
Mediational analysis 1: MOS-SSS							
β	-0.656	2.107	-9.613	-8.230	-1.383*	0.143	0.302
LLCI	-0.828	0.892	-11.250	-10.027	-2.285		
ULCI	-0.484	3.321	-7.977	-6.433	-0.564		
Mediational analysis 2: Emotional/informational support							
β	-0.708	2.161	-9.613	-8.082	-1.531*	0.159	0.311
LLCI	-0.903	1.167	-11.250	-9.836	-2.365		
ULCI	-0.513	3.155	-7.977	-6.327	-0.783		
Mediational analysis 3: Affectionate support/positive social interaction							
β	-0.648	2.056	-9.587	-8.254	-1.333*	0.139	0.300
LLCI	-0.796	0.772	-11.224	-10.091	-2.258		
ULCI	-0.499	3.341	-7.950	-6.416	-0.483		
Mediational analysis 4: Tangible support							
β	-0.589	1.088	-9.635	-8.993	-0.641*	0.066	0.286
LLCI	-0.771	0.131	-11.272	-10.713	-1.294		
ULCI	-0.407	2.045	-7.998	-7.274	-0.090		

*Significant association, N.B: Models adjusted for age, highest qualification, and marital status. Abbreviation: LLCI: Lower Limit Confidence Interval; ULCI: Upper-Limit Confidence Interval

The mediational analysis revealed a significant indirect effect on HRQOL-mental health domain (MCS) by perceived stress through social support (β = -1.38; 95% CI: -2.28, -0.56), and the mediating variable accounted for around 14.3% of the total effect. The R^2^ med value of 0.302 shows that 30.2% of the variance in MCS was due to the indirect effect of perceived stress through overall social support.

A mediational analysis by specific domains of social support as a mediator found a significant mediation effect in all three components. There was a significant indirect effect of perceived stress through emotional/informational support (β= **-**1.53; 95% CI: -2.36, -0.78), tangible support (β= -0.64; 95% CI: -1.29, -0.09) and affectionate support (β= **-**1.33; 95% CI: -2.25, -0.48). The mediating variables, emotional/informational support, tangible support and affectionate support, accounted for 15.9, 6.6 and 13.9% of the total effect, respectively. These results revealed that overall social support and domains of social support partially mediated the relationship between perceived stress and MCS.

### The direct effect of social support on HRQOL (i.e. MCS and PCS)

The results of the multivariate QR analysis examining the direct effects of social support on HRQOL are displayed in Table 4. After adjusting for confounders, the β-estimates indicated that all domains of social support and overall social support scores were positively associated with higher MCS scores (p < 0.05). Conversely, after adjusting for confounders, social support was found to have no significant association with PCS (p > 0.05).

**Table 4:** Multivariate QR model examining the association between social support and HRQoL (MCS and PCS).

Social support		Mental Component Score (MCS)		Physical Component Score (PCS)	
		Model I (β coefficient, 95% CI)	Model II^‡^ (β coefficient, 95% CI)	Model I (β coefficient, 95% CI)	Model II^¥^ (β coefficient, 95% CI)
Emotional/Informational support	25th Quantile	5.28 (3.91, 6.65)^**^	3.05 (1.97, 4.12)^**^	1.41 (-0.45, 3.29)	-1.34 (-2.97, 0.30)
	50th Quantile	3.53 (2.65, 4.40)^**^	1.23 (0.36, 2.09)^*^	1.82 (0.13, 3.51)^*^	-0.06 (-1.30, 1.18)
	75th Quantile	2.33 (1.54, 3.12)^**^	1.38 (0.57, 2.21)^*^	0.39 (-0.51, 1.30)	0.62 (-0.54, 1.78)
	90th Quantile	1.87 (0.95, 2.79)^**^	1.32 (0.59, 2.06)^**^	0.04 (-0.84, 0.93)	0.54 (-0.27, 1.35)
Affectionate support/ positive social interaction	25th Quantile	5.39 (3.91, 6.87)^**^	3.15 (1.68, 4.62)^**^	1.43 (-0.69, 3.57)	-0.04 (-2.0, 1.91)
	50th Quantile	4.20 (3.07, 5.33)^**^	1.17 (0.08, 2.26)^*^	2.65 (0.55, 4.74)^*^	-0.20 (-1.79, 1.38)
	75th Quantile	2.59 (1.65, 3.54)^**^	0.15 (-0.82, 1.13)	0.81 (-0.30, 1.92)	0.96 (-0.42, 2.34)
	90th Quantile	2.08 (0.95, 3.21)^**^	1.02 (0.183, 1.86)^*^	0.16 (-0.93, 1.25)	0.52 (-0.39, 1.44)
Tangible support	25th Quantile	3.33 (2.05, 4.61)^**^	1.77 (0.65, 2.88)^*^	1.17 (-0.61, 2.95)	-0.98 (-2.47, 0.51)
	50th Quantile	3.06 (2.14, 3.97)^**^	0.54 (-0.25, 1.34)	1.15 (-0.55, 2.86)	-0.57 (-1.75, 0.59)
	75th Quantile	1.15 (0.44, 1.86)^*^	-0.36 (-1.10, 0.37)	0.68 (-0.19, 1.56)	0.22 (-0.81, 1.24)
	90th Quantile	1.01 (0.09, 1.92)^*^	-0.14 (-0.84, 0.55)	0.23 (-0.65, 1.13)	0.44 (-0.29, 1.18)
Overall social support	25th Quantile	5.54 (4.05, 7.02)^**^	2.71(1.43, 3.97)^**^	1.36 (-0.70, 3.42)	-1.15 (-2.94, 0.62)
	50th Quantile	3.88 (2.89, 4.88)^**^	1.41 (0.42, 2.39)^*^	1.96 (-0.02, 3.95)	-0.45 (-1.92, 1.01)
	75th Quantile	2.29 (1.38, 3.20)^**^	0.43 (-0.50, 1.37)	0.88 (-0.14, 1.92)	1.07 (-0.25, 2.40)
	90th Quantile	1.86 (0.82, 2.91)^**^	0.94 (0.19, 1.68)^*^	0.14 (-0.89, 1.17)	0.69 (-0.14, 1.52)

*p < 0.05; **p < 0.001

^‡^Adjusted for: age, marital status, residence, highest educational qualification, able to manage on income available, BMI, gestational age, parity, alcohol consumption and perceived stress;

^¥^Adjusted for age, marital status, residence, highest educational qualification, able to manage on income available, BMI, gestational age, parity, alcohol consumption, perceived stress and physical activity

Specifically, emotional/informational support was found to have a statistically significant association with MCS in all four quantiles, highest in the 25th quantile (β = 3.05; 95% CI: 1.97, 4.12) and least in the 90th quantile (β = 1.32, 95% CI: 0.59, 2.06). Furthermore, affectionate support/positive social interaction a significantly associated with MCS in all three quantiles (p < 0.05) except the 75th quantile (β = 0.15, 95% CI: -0.82, 1.13). At the 25th quantile, the adjusted QR model also indicated a significant (p < 0.05) but a 47% reduction in the magnitude of the association between tangible support and MCS (model I vs. model II). However, tangible support was not significantly associated with the remaining 50th, 75th, and 90th quantiles (p > 0.05).

Overall social support has a significant direct effect on MCS at the 25th (β = 2.71, 95% CI: 1.43, 3.97), 50th (β = 1.41, 95% CI: 0.42, 2.39) and 90th quantiles (β = 0.94, 95% CI: 0.19, 1.68). But the magnitude of association decreased as the distribution of MCS scores changed from the 25th to the 90th quantile.

## Discussion

This study - examining the direct effect of social support on HRQoL as well as the mediating effects of social support in the relationship between perceived stress and HRQoL during pregnancy - reveals several important findings. This study supplements limited evidence on the topic and presents the first study to examine the mediating effect of social support in the relationship between perceived stress and HRQoL among pregnant women.

Our results illustrate that overall social support and all three domains of social support have a significant positive association with the HRQoL-mental health domain (MCS) and are negatively associated with perceived stress during pregnancy. In addition, overall social support and all three domains of social support play a significant partial mediational role in the relationship between perceived stress and the HRQoL-mental health domain (MCS). It has been shown that social support plays a similar partial mediating effect in the association between perceived stress and HRQoL among Cancer [63], and HIV/AIDS patients [64], Chinese Shidu parents [65] and earthquake survivors [66]. The partial mediating effect of social support is explained by the stress-buffering hypothesis, which suggests that social support contributes to the well-being of individuals by enhancing positive affect, stress coping ability and perceived self-worth of individuals, which indirectly helps to improve the HRQoL of pregnant women [42].

Our study also found that social support is positively associated with the HRQoL-mental health domain. Previous studies also found a significant positive association between social support and HRQoL [67–70]. For example, a longitudinal study conducted in Australia among a sample of women (n = 473) found that social support was a significant predictor of HRQoL-mental health domain during pregnancy and after childbirth [67]. A study by Vahideh et al. (2016) among 477 Hungarian pregnant women also found that social support had a significant association with better HRQoL both in nulliparous and multiparous women [68]. Further, a cross-sectional study conducted in China among a sample of pregnant women (n = 267) also reported the significant direct effect of social support on the mental health component of HRQoL [69]. However, none of the above studies examined the direct effect of specific domains of social support (i.e., emotional/informational, affectionate, tangible or instrumental support) on HRQoL, which makes our study more robust.

However, the current study also found a non-significant association between social support and HRQoL-physical domain (PCS). A similar finding was reported by a study conducted in Australia (n = 473), where social support did not significantly predict the HRQoL-physical domain during pregnancy or following childbirth [67]. The study by Emmanuel et al. selected pregnant women from three public hospitals in metropolitan Brisbane, Australia, and it employed SF-12 and Maternal Social Support Scale (MSSS) to examine HRQoL and social support, respectively, at 36 weeks of pregnancy and 6 and 12 weeks after giving birth [67].

The findings from our study suggest that to overcome the challenges of stress during pregnancy, it is important to integrate social support as an intervention strategy targeting pregnant women suffering from stress. There are two reasons why social support intervention is important. First, social isolation could prevent pregnant women from getting help and other services from the available social support network [71], which can exacerbate mental health problems and worsen the HRQoL. Second, social support interventions can enhance adherence to other recommended treatments, which helps to improve the subjective feeling of pregnant women, reducing stress and other psychological problems, and as a result, improving the QoL [71]. Pregnant women who receive adequate social support pay significant attention to pregnancy-related changes, which in turn could inspire them to engage in good pregnancy care practices [72]. A strong support network helps in improving the HRQoL of pregnant women, thereby protecting pregnant women against stress [73] or providing more favourable socioeconomic conditions [74]. Conversely, evidence also indicated that low social support significantly predicts health risk factors such as decreased physical activity [75], increased alcohol intake [75], and high BMI [76], which leads to deteriorated HRQoL.

Therefore, such significant effects of social support on HRQoL during pregnancy have an implication for policy and clinical practice. First, maternal health professionals need to consider social support as an essential tool to improve the HRQoL of pregnant women, and there should be routine awareness creation work on the importance of social support during pregnancy. Second, as part of routine antenatal care activity, it is beneficial to integrate screening tools for assessing the level of social support when recording the medical history of pregnant women during an antenatal care visit. Third, further psychological counselling and incorporating stress management as an intervention would help reduce stress and its subsequent effect on pregnant women’s mental and physical health. Fourth, policymakers should work towards establishing community-based social support programs to enhance the wider community’s awareness about the role of social support (i.e., support from spouse, family or peer) in improving the well-being of pregnant women. Finally, future longitudinal studies are recommended to explore the causative relationship between social support and HRQoL over different periods of pregnancy.

## Limitations

Some limitations need attention when making inferences from our findings. Firstly, the study depends on self-reported data from study participants, which has the potential to introduce recall bias. Second, our study mainly focused on the linkage between perceived stress, social support, and HRQoL. Therefore, other studies are advised to explore additional mediating variables in the relationship between perceived stress and HRQoL. Third, since our study employed a cross-sectional design, causal relationships between the examined variables cannot be determined. Fourth, our findings are limited to pregnant women within the age range of 34–39 years. As such, any interpretation of our findings concerning younger pregnant women must be undertaken with caution.

## Conclusion

Our study has shown that social support plays a direct and mediating role in improving the HRQoL-mental health of pregnant Australian women. Thus, social support can play a role in helping reduce the effects of stress, which in turn improves the HRQoL-mental health during pregnancy. Therefore, maternal health professionals need to consider social support as an essential tool to improve the HRQoL of pregnant women. Also, as part of routine antenatal care activity, it is beneficial to assess the level of social support of pregnant women.

## Electronic supplementary material

Below is the link to the electronic supplementary material.

**Additional file 1**

**Additional file 2**

## Data Availability

ALSWH survey data are owned by the Australian Government Department of Health and due to the personal nature of the data collected, release by ALSWH is subject to strict contractual and ethical restrictions. De-identified data are available where a formal request to make use of the material has been approved by the ALSWH Data Access Committee. The committee is receptive of requests for datasets required to replicate results. Information on applying for ALSWH data is available from https://alswh.org.au/for-data-users/applying-for-data/ Also, the Corresponding Author can be contacted if someone wants to request the data from this study.

## Abbreviations

ALSWH: Australian Longitudinal Study on Women’s Health
CI: Confidence Interval
HRQoL: Health-Related Quality of Life
MCS: Mental component Scale
MOS-SSS-19: 19 item Medical Outcome Study Social Support Scale
PCS: Physical Component Scale
QoL: Quality of Life
SD: Standard Deviation
SPSS: Statistical Package for Social Science
VIF: Variance Inflation Factor
WHO: World Health Organization
