# Supplementary material for: The direct and mediating effect of social support on health-related quality of life during pregnancy among Australian women
Source: BMC Pregnancy Childbirth. 2023 May 22;23:372. doi: 10.1186/s12884-023-05708-0 (PMC10204163; doi:10.1186/s12884-023-05708-0)
Supplement: Supplementary file 2 — Additional file 2 [file 12884_2023_5708_MOESM2_ESM.docx]

**Supplementary file 2:** Mean and median of domains of SF-36 (N=493).

| Domain of SF-36 | Mean ±SD | Median |
| --- | --- | --- |
| Physical functioning | 80.11 (20.66) | 90.00 |
| Role physical | 63.29 (41.85) | 75.00 |
| Role emotional | 90.06 (25.37) | 100.00 |
| Bodily pain | 71.60 (20.45) | 74.00 |
| Vitality | 52.22 (18.02) | 55.00 |
| Mental health | 77.61 (13.80) | 80.00 |
| Social functioning | 80.86 (21.36) | 87.50 |
| General health | 77.84 (15.79) | 80.00 |
| MCS | 50.48 (8.09) | 52.54 |
| PCS | 46.79 (9.69) | 49.10 |

Abbreviation: MCS: Mental Component score, PCS: Physical Component Score.
